# Reduced or modified dietary fat for preventing cardiovascular disease

**DOI:** 10.1590/1516-3180.20161342T1

**Published:** 2015-03-17

**Authors:** Lee Hooper, Carolyn D. Summerbell, Rachel Thompson, Deirdre Sills, Felicia G. Roberts, Helen J. Moore, George Davey Smith

## Abstract

**BACKGROUND:**

Reduction and modification of dietary fats have differing effects on cardiovascular risk factors (such as serum cholesterol), but their effects on important health outcomes are less clear.

**OBJECTIVE:**

To assess the effect of reduction and/or modification of dietary fats on mortality, cardiovascular mortality, cardiovascular morbidity and individual outcomes including myocardial infarction, stroke and cancer diagnoses in randomised clinical trials of at least 6 months duration.

**METHODS:**

*Search methods:* For this review update, the Cochrane Central Register of Controlled Trials (CENTRAL), Medline and Embase, were searched through to June 2010. References of Included studies and reviews were also checked.

*Selection criteria:* Trials fulfilled the following criteria: 1) randomized with appropriate control group, 2) intention to reduce or modify fat or cholesterol intake (excluding exclusively omega-3 fat interventions), 3) not multi factorial, 4) adult humans with or without cardiovascular disease, 5) intervention at least six months, 6) mortality or cardiovascular morbidity data available.

*Data collection and analysis:* Participant numbers experiencing health outcomes in each arm were extracted independently in duplicate and random effects meta-analyses, meta-regression, sub-grouping, sensitivity analyses and funnel plots were performed.

**MAIN RESULTS:**

This updated review suggested that reducing saturated fat by reducing and/or modifying dietary fat reduced the risk of cardiovascular events by 14% (RR 0.86, 95% CI 0.77 to 0.96, 24 comparisons, 65,508 participants of whom 7% had a cardiovascular event, I2 50%). Subgrouping suggested that this reduction in cardiovascular events was seen in studies of fat modification (not reduction - which related directly to the degree of effect on serum total and LDL cholesterol and triglycerides), of at least two years duration and in studies of men (not of women). There were no clear effects of dietary fat changes on total mortality (RR 0.98, 95% CI 0.93 to 1.04, 71,790 participants) or cardiovascular mortality (RR 0.94, 95% CI 0.85 to 1.04, 65,978 participants). This did not alter with sub-grouping or sensitivity analysis. Few studies compared reduced with modified fat diets, so direct comparison was not possible.

**AUTHORS' CONCLUSIONS:**

The findings are suggestive of a small but potentially important reduction in cardiovascular risk on modification of dietary fat, but not reduction of total fat, in longer trials. Lifestyle advice to all those at risk of cardiovascular disease and to lower risk population groups, should continue to include permanent reduction of dietary saturated fat and partial replacement by unsaturates. The ideal type of unsaturated fat is unclear.

The abstract is available free-of-charge from: http://onlinelibrary.wiley.com/doi/10.1002/14651858.CD002137.pub3/abstract.
